# Progesterone receptor membrane component 1 inhibits tumor necrosis factor alpha induction of gene expression in neural cells

**DOI:** 10.1371/journal.pone.0215389

**Published:** 2019-04-26

**Authors:** Karlie A. Intlekofer, Kelsey Clements, Haley Woods, Hillary Adams, Alexander Suvorov, Sandra L. Petersen

**Affiliations:** 1 Department of Veterinary and Animal Sciences, Institute of Applied Life Sciences, University of Massachusetts Amherst, Amherst, Massachusetts, United States of America; 2 Department of Environmental Health Sciences, University of Massachusetts Amherst, Amherst, Massachusetts, United States of America; National Institutes of Health, UNITED STATES

## Abstract

Progesterone membrane receptor component 1 (Pgrmc1) is a cytochrome b5-related protein with wide-ranging functions studied most extensively in non-neural tissues. We previously demonstrated that Pgrmc1 is widely distributed in the brain with highest expression in the limbic system. To determine Pgrmc1 functions in cells of these regions, we compared transcriptomes of control siRNA-treated and Pgrmc1 siRNA-treated N42 hypothalamic cells using whole genome microarrays. Our bioinformatics analyses suggested that Pgrmc1 plays a role in immune functions and likely regulates proinflammatory cytokine signaling. In follow-up studies, we showed that one of these cytokines, TNFα, increased expression of *rtp4*, *ifit3 and gbp4*, genes found on microarrays to be among the most highly upregulated by Pgrmc1 depletion. Moreover, either Pgrmc1 depletion or treatment with the Pgrmc1 antagonist, AG-205, increased both basal and TNFα-induced expression of these genes in N42 cells. TNFα had no effect on levels of Rtp4, Ifit3 or Gbp4 mRNAs in mHippoE-18 hippocampal control cells, but Pgrmc1 knock-down dramatically increased basal and TNF*α*-stimulated expression of these genes. P_4_ had no effect on *gbp4*, *ifit3* or *rtp4* expression or on the ability of Pgrmc1 to inhibit TNFα induction of these genes. However, a majority of the top upstream regulators of Pgrmc1 target genes were related to synthesis or activity of steroids, including P_4_, that exert neuroprotective effects. In addition, one of the identified Pgrmc1 targets was Nr4a1, an orphan receptor important for the synthesis of most steroidogenic molecules. Our findings indicate that Pgrmc1 may exert neuroprotective effects by suppressing TNFα-induced neuroinflammation and by regulating neurosteroid synthesis.

## Introduction

Progesterone receptor membrane component 1 (Pgrmc1) is an ancient and somewhat enigmatic molecule with a diverse range of functions and multiple intracellular locations. Structurally, it is a 28-kDa protein with an N-terminal extracellular region, a single transmembrane domain and a cytoplasmic region. The cytoplasmic region contains a cytochrome b5-like heme-binding domain that allows interaction with a number of steroidogenic and drug-metabolizing cytochrome P450 enzymes [1–4]. As suggested by its name, Pgrmc1 also contains a high-affinity progesterone (P_4_) binding site [5–9], most likely within the transmembrane domain and the initial segment of the C terminus [10] and near the heme-binding site [7]. Finally, the Pgrmc1 molecule contains sites that may allow interaction with SH2- and SH3-domain signaling proteins [11].

In view of these diverse molecular structural features, it is not surprising that Pgrmc1 has been implicated in such wide-ranging functions as steroid synthesis [4], heme sensing and Ssynthesis [12], regulation of fatty acid 2 hydroxylase [13], stabilization of tyrosine kinase receptors in cell membranes [14, 15], suppression of p53 and Wnt/β-catenin pathways [16], inhibition of ovarian granulosa cell apoptosis [17] and promotion of breast cancer cell survival and tumor growth [18]. Most studies delineating Pgrmc1 functions have been performed in non-neural tissues and cell lines, but this molecule is also widely distributed throughout the brain [19–22]. Unfortunately, information about Pgrmc1 in neural cells is rather sparse and no unifying concept regarding its neural functions or signaling pathways has emerged.

To better understand the molecular and cellular functions of Pgrmc1 in neural cells, we compared transcriptomes of hypothalamic N42 cells with and without Pgrmc1 knockdown. We independently verified our findings using QPCR and used several bioinformatics tools to identify pathways and neural processes likely regulated by Pgrmc1. Results of these analyses suggest that Pgrmc1 blocks expression of genes downstream of proinflammatory cytokines in a P_4_-independent manner.

## Materials and methods

### Cells and culturing methods

Immortalized hypothalamic N42 neuronal cells (CELLutions Biosystems, Inc.; Burlington, Ontario, Canada; [23]) and mEH-18 hippocampal neuronal cells (CELLutions Biosystems, Inc., [24]) were used for these studies. We verified with PCR that cells of this line do not express the canonical progesterone receptor (Pgr) (see S1 Fig).

Cells were maintained at 37° C and 5% CO_2_ in Dulbecco’s Modified Eagle Medium (DMEM) supplemented with 10% (v/v) fetal bovine serum (Hyclone FBS; Thermo Fisher Scientific, Rockford, IL), 100 U/ml penicillin, 100 mg/ml streptomycin, and 2 mM L-glutamine (PS-Gln; GIBCO-BRL; Gaithersburg, MA). When cells reached 60–70% confluence, they were rinsed with phosphate buffered saline (PBS) and media was replaced with phenol red-free DMEM with 10% charcoal-stripped FBS and PS-Gln. After 24 h, cells were transfected with Pgrmc1 siRNA or control scramble sequence constructs.

### Pgrmc1 knockdown with siRNA

N42 cells were transfected with 2 μM negative control or Pgrmc1 siRNA constructs (Cat no. S184526 and SI03650318, ThermoFisher; Waltham, MA), using HiPerfect Transfection Reagent according to the manufacturer’s protocol (Qiagen; Valencia, CA). Cells were incubated with siRNA for 24 h before harvesting.

To verify Pgrmc1 knockdown, we performed western blots on protein isolated from N42 cell lysates using radioimmunoprecipitation assay buffer (Boston BioProducts; Worcester, MA) containing 1% protease inhibitor cocktail (Calbiochem; San Diego, CA). Protein concentrations were assessed using Pierce BCA Protein Assay (Thermo Fisher Scientific). Ten μg of protein per lane was electrophoresed on 4–15% Tris-HCl SDS-PAGE gradient gels (Biorad; Hercules, CA) and was transferred electrophoretically to Immobilon-P membranes (EMD Millipore; Billerica, MA). Membranes were blocked in 5% skimmed dry milk in Tris-buffered saline plus 0.05% Tween-20 (TBST; pH 7.4) for 1 h at room temperature on an orbital shaker, and then incubated overnight at 4° C with anti-Pgrmc1 antibody (1:1000; Proteintech Group; Chicago, IL) in TBST. Membranes were washed in TBST for 30 min, followed by incubation with horseradish peroxidase-conjugated secondary antibody (anti-rabbit 1:10,000; Abcam; Cambridge, MA) for 1 h, and developed using Chemiluminescence HRP Substrate (EMD Millipore). Blots were apposed to BioMax MR film (Kodak; Rochester, NY), and band intensities were quantified from developed films using densitometric software (GeneTools ver. 3.07 SynGene; Cambridge, England). Blots were re-probed with anti-β-actin (1:2000; Abcam) and anti-mouse antisera (1: 2000; Abcam) to provide protein loading controls.

We also used QPCR to examine effects of Pgrmc1 knockdown on Pgrmc1 mRNA levels in N42 cells. We isolated RNA using the RNeasy Mini Kit (Qiagen) and reverse-transcribed using the QuantiTect Reverse Transcription Kit (Qiagen). QPCR was performed in a Stratagene Mx3000P thermocycler (Agilent Technologies; Wilmington, DE) programmed as follows: 95^o^ C, 10 min; 40 cycles of 95^o^ C for 15 sec; 60^o^ C for 60 sec; and 72^o^ C for 60 sec. We used the QuantiTect SYBR Green Kit (Qiagen) for QPCR following the manufacturer’s protocol.

We determined primary efficiency over a range of cDNA concentrations and included samples with no cDNA as negative controls. Primer specificity was validated by observing a single fluorescence peak in each QPCR reaction and also using 2% agarose gel electrophoresis to verify that single products were obtained following the reaction. Further verification entailed a melting curve analyses in which samples were heated to 95^o^ C and fluorescence measurements recording at incremental increases of 0.5^o^ C for 80 cycles.

Data for each sample and controls were obtained using MxPro QPCR analysis software (Agilent Technologies). We used the ΔΔCt method to compare treatment and control samples [25], converting data to percent control in each pairwise comparison and calculating means for treatment groups (Pgrmc1 siRNA vs scramble control siRNA). Means of treatment pairs were compared using student t-tests.

### Affymetrix microarray analysis

For these studies, we chose to use microarray technology rather than RNA-Seq because the former is better suited for detecting genes with relatively low expression levels [26]. RNA from N42 cell siRNA experiments described above was used for Mouse Genome 430 2.0 Gene Chip assays (Affymetrix; Palo Alto, CA) performed by the Keck Microarray Institute at Yale University. RNA quality was assessed using an Agilent 2100 Bioanalyzer and RNA 6000 Nano LabChips (Agilent Technologies).

For each treatment group (cells transfected with Pgrmc1 siRNA or with control scramble siRNA), we used three pools of RNA extracted from three replicate studies of transfected N42 cells. The quality of total RNA in each sample was assessed using a Nanodrop Spectrophotometer (Thermo Scientific; Wilmington, DE) and a 2100 Agilent Bioanalyzer (Agilent Technologies). Samples were accepted for analysis if the 260/280 ratio was at least 1.8 and the RNA Integrity Number was greater than 8.0. Preparation of labeled cRNA for hybridization onto Affymetrix GeneChips followed the recommended Affymetrix protocol.

The Yale Center for the NIH Neuroscience Microarray Consortium carried out microarray analysis. Double-stranded cDNA was synthesized from 1 to 5 μg of total RNA using a Superscript Choice System (Life Technologies; Carlsbad, CA) with an HPLC-purified oligo (dT) primer containing a T7 RNA polymerase promoter sequence at the 5’-end (Proligo LLC; Boulder, CO). We synthesized the second cDNA strand using *E*. *Coli* DNA polymerase I, RNase H and DNA ligase, and then generated labeled cRNA using a GeneChip IVT labeling kit (Affymetrix) following the manufacturer’s instructions. The labeling procedure incorporated biotinylated synthetic analogs by using a pseudouridine reagent and MEGAscript T7 RNA Polymerase (Life Technologies). Biotin-labeled cRNA was purified using GeneChip cleanup module (Affymetrix). The cRNA was incubated at 94^o^ C for 35 min in fragmentation buffer and the resulting 35- to 200-base fragments were hybridized to the arrays for 16 h at 45^o^ C.

After hybridization, we washed arrays using an Affymetrix fluidics station and stained them with streptavidin-phycoerythrin (10 μg/ml; Life Technologies). Arrays were inspected for hybridization artifacts and then scanned with an Affymetrix GeneChip Scanner 3000. Images were analyzed using Affymetrix Microarray Suite 5.0, scaling to a target average intensity. Quality controls included sense strand probes and three housekeeping genes (*gapdh*, *hexokinase* and *β-actin*). We also evaluated spiked controls, background values, scanner noise (Q value) and scaling factors. The CEL files containing levels of probe intensities were analyzed with Partek Genomics Suite, version 6.15 (Partek; St Louis, MO) with the Robust Multi-array Average method of normalization. *P* values were corrected for multiple hypothesis testing using the Benjamini-Hochberg method to control for false discovery. Finally, we performed hierarchical clustering analyses of genes regulated by at least 1.2-fold with *p*<0.05.

### Interrogation of microarray data

We used multiple bioinformatics strategies to identify P_4_-independent pathways likely regulated by Pgrmc1 in neural cells. Integrating data generated with tools that use different algorithms and databases allowed us to develop a more comprehensive mechanistic model.

#### Identification of the most highly regulated genes

We first identified genes regulated by at least 1.2-fold and with a significance of *p*<0.05 in each of the two comparison groups (S2 Dataset). We then used GeneCards (www.genecards.org) and NIH Gene (www.ncbi.nlm.nih.gov/gene) to determine general functions of these most highly regulated genes.

#### Pathway analysis

We compared the transcriptomes of Pgrmc1 siRNA- and scramble siRNA-treated N42 cells with Gene Set Enrichment Analysis (GSEA; http://www.broadinstitute.org/gsea/index.jsp), software that allows detection of sets of affected genes overrepresented in specific pathways without consideration of the fold change of genes induced by treatment. We used the Hallmark Gene Set from the Molecular Signatures Database of genes known to be involved in specific biological or biochemical relationships or shown to be co-expressed or co-regulated. The Hallmark Gene Sets include 50 sets of genes that represent well characterized biological processes (http://www.broadinstitute.org/gsea/msigdb/collections.jsp).

We also compared transcriptomes of Pgrmc1 siRNA-treated and control cells using Ariadne Pathway Studio (www.elsevier.com/solutions/pathway-studio-biological-research). The Pathway Studio program is based on a manually curated database of biological relationships derived from published articles beyond the public domain [27].

#### Ingenuity upstream regulator analysis

We used the Ingenuity Pathway Analysis (Qiagen; https://www.qiagenbioinformatics.com/blog/discovery/publication-roundup-ingenuity-pathway-analysis-3 to identify upstream transcriptional regulatory cascades linked to the gene changes observed in response to Pgrmc1 knockdown in the absence or presence of P_4_. For this analysis, we selected the genes that changed by at least 1.2-fold and at a significance level of *p*<0.01.

#### Database for Annotation, Visualization and Integrated Discovery (DAVID) analysis

We used DAVID Bioinformatics Resources 6.8 program [28, 29] to functionally categorize genes into clusters and determine the fold-enrichment of genes in these clusters. DAVID uses different databases than GSEA and simplifies the process of determining the likely biological significance of microarray data by integrating gene identifier and functional category terms from multiple bioinformatics databases. DAVID uses different databases than GSEA, including Kyoto Encyclopedia of Genes and Genomes, Clusters of Orthologous Groups, Gene Ontology Consortium and the PANTHER Classification System.

DAVID converted the data to DAVID IDs and removed redundancies so that genes detected with multiple probes were not overrepresented in the analysis. We used Functional Annotation Clustering analysis with default databases and stringency settings except that we increased the Kappa Similarity Overlap to 3, Similarity Threshold to 0.7 and the Final Group Membership to 3 in order to minimize duplication of genes in different clusters.

### QPCR verification of microarray findings

We used QPCR to verify changes in gene expression of targets identified in our microarray analysis. For QPCR studies, we transfected N42 cells with scrambled control siRNA or Pgrmc1 siRNA as described for microarray studies. We then isolated mRNA, performed reverse transcription, measured mRNA levels and verified Pgrmc1 knockdown using QPCR and western blots as described above. Primer pairs for gene targets (Table 1) were based on sequences from the Affymetrix Mouse Genome 430 2.0 Gene Chips and were obtained from Integrated DNA Technologies (Coralville, Iowa). To detect spectrin alpha (Spta1) mRNA, we used pre-validated primers (Cat. No. 32969A; SABiosciences). Primary efficiency and specificity were verified as described above.

**Table 1:** Primers used for QPCR validation of targets identified on microarrays to be significantly regulated by Pgrmc1 knockdown.

Gene Target	Primer Pairs
***Etv1***	3' AATGAGGGCCACCGTTTTGG
	5' CCAAAACAGCAGGATGGCAC
***Gbp4***	3’ CTGTGCTGTGGGACCAGATT
	5’ TGGCTTCCTGGTTCACTGAC
***Gpnmb***	3' TGTCCTGATCTCCATCGGCT
	5' TGGCTTGTACGCCTTGTGTT
***Ifit3***	3' ACTCTTTGGTCATGTGCCGT
	5' AGGACTTCGCCTCCTCTGAA
***Nr4a1***	3’ TTCCCACCACCAGCCACCCA
	5’ CTCGCTGCCACCTGAAGCCC
***Pgrmc1***	3' CCTCTGCATCTTCCTGCTCTA
	5' CGAGCTGTCTCGTCTTTTGG
***Rtp4***	3' AGGCACGCATGAGGATCTTT
	5' CCAAAGTCACCTTCTCACCCA
***Tgtp***	3' CCCTAAGAGGAAAGCCATCACA
	5' TGGCTCTGTATGGTAGAAGCTC
***Trim5***	3' GCTGGTTCAAAACAACAATCCA
	5' GCCATGTTCAAGATTCCTTGCTT
***Usp18***	3' GCTTGACTCCGTGCTTGAGA
	5' CCCAAACCCCTTGCCCATTA
***Utx***	3' TTGTCGCCTTTAGAACCTTGG
	5' ATGGTAAGACAAAGGGCACAGA

Data for each sample and controls were obtained using MxPro QPCR analysis software (Agilent Technologies). We used the ΔΔCt method to compare treatment and control samples [25], converting data to percent control in each pairwise comparison and calculating means for treatment groups (Pgrmc1 siRNA vs scramble control siRNA). Means of treatment pairs were compared using student t-tests.

### Examination of Pgrmc1 antagonist and P_4_ effects on expression of selected genes

To determine whether administration of the Pgrmc1 antagonist, AG-205, altered expression of genes found to be regulated by Pgrmc1, cells were seeded at a density of 50,000 cells/well in 24-well plates and allowed to grow for 48 h in 5% CO_2_ at 37^o^ C. They were treated with 10 μM AG-205 (Sigma; optimal dosage determined in preliminary studies) or vehicle (cell culture grade DMSO) for 24 h. To determine whether these genes were also regulated by P_4_, we treated N42 cells with either P_4_ (10 or 100 nM) or vehicle for 8 h. RNA was then isolated, reverse transcribed and the cDNA was then used as a template in QPCR studies as described above.

### Examination of Pgrmc1 effects on TNFα induction of genes

#### Effects of Pgrmc1 knockdown or AG-205 on TNFα-induced expression of genes

We tested whether TNFα affects *ifit3*, *gbp4 and rtp4* gene expression and whether Pgrmc1 depletion or blockade changes the responses to TNFα. We chose these targets because they were among the genes most highly regulated by Pgrmc1 knockdown, and they are known targets of cytokines [30–33]. To deplete cells of Pgrmc1, we transfected N42 cells with 10 nM Pgrmc1 siRNA or negative control siRNA, and 40 h later treated cells with 10 ng/ml (0.59 nM) TNFα for 8 h as described above. In a separate study, we treated N42 cells with 10 μM AG-205 as described above, incubating the cells for 24 h. RNA was isolated, reverse-transcribed and cDNA used for measurement of Ifit3, Gbp4 and Rtp4 mRNAs.

#### Effects of Pgrmc1 overexpression on TNFα-induced expression of genes

To determine the effects of overexpression of Pgrmc1 on TNFα-dependent gene expression, we constructed a Pgrmc1 expression vector with puromycin resistance using PCR. We amplified the mouse Pgrmc1 coding sequence with primers that also contained regions homologous to the PstI restriction enzyme cut site region on the 5’ ends. The forward primer was: AAC CGG ATC CTC TAG AGT CGA TGG CTG CCG AGG ATG TGG TGG CG and the reverse primer was: CCC CAA GCT TGC ATG CCT GCT CAT TCA TTC TTC CGA GCT GTC. The pBApo-CMV Pur plasmid (Clontech) was digested with PstI (Promega) and ligated to the Pgrmc1 PCR product using the NEBuilder HiFi DNA Assembly Master Mix (New England Biolabs; Ipswich, MA). We then transformed competent *E*. *coli* with the resulting Pgrmc1 expression vectors and isolated the plasmids using a Qiagen Plasmid Midi Kit. We confirmed correct insertion of the full coding sequence of Pgrmc1 by sequencing. To generate the stable cell line, N42 cells were plated at a density of 200,000 cells/well in 6-well plates. The following day, cells were transfected with Pgrmc1 expression vector or empty vector using Genejuice transfection reagent (EMD Millipore) following manufacturer’s directions. After 48 h, cells were trypsinized and split 1:4 in DMEM containing 3 μg/ml puromycin hydrochloride (Cayman Chemical; Ann Arbor, MI). Puromycin-containing media was changed every three days to allow for selection of stably-expressing cells. We verified overexpression of Pgrmc1 with western blots using the antibody and procedures described above in studies verifying Pgrmc1 downregulation by siRNA.

The resulting N42 cells stably expressing empty or Pgrmc1-containing vector were plated in 24-well plates at a density of 25,000 cells/well and grown in DMEM containing 1 μg/ml puromycin. Forty-eight h later, the cells were treated with 10 ng/ml mouse TNFα (Invitrogen) or vehicle (sterile 0.5% BSA in PBS) for 8 h. After lysing the cells in TRIzol (Invitrogen), RNA was extracted, reverse-transcribed and used in QPCR analyses as described above.

For these studies, means were compared among groups using Two-Way ANOVA with Pgrmc1 and TNFα treatments as main effects. Tukey’s multiple comparison test was used for *post hoc* analyses.

#### Effects of P_4_ on TNFα-induced expression of target genes

Considering that P_4_ is a ligand of Pgrmc1, we tested whether TNFα-induction of *ifit3*, *gbp4* and/or *rtp4* expression was affected by P_4_ as it is by Pgrmc1 manipulations_._ We cultured N42 cells as described above, then treated with either P_4_ (10 or 100 nM) or vehicle for 1 h before treating with TNFα or vehicle for 8 h. RNA was then isolated using TRIzol (Invitrogen) using the manufacturer’s protocol. RNA was reverse transcribed with MMLV-RT (Promega) in the presence of RNAsin (Promega) to prevent RNA degradation. The resulting cDNA was then used as a template in QPCR studies using the appropriate gene-specific primers (Table 1). Data were analyzed as described above.

#### Effects of Pgrmc1 on cytokine-induced expression of target genes in a hippocampal cell line

To determine whether the effects of Pgrmc1 on expression of gene targets of pro-inflammatory cytokines is similar in hippocampal cell lines, we used mHippoE-18 cells (CELLutions Biosystems, Inc.). Cells were grown, transfected with Pgrmc1 or negative control siRNA and treated with TNFα as described above. RNA was extracted, reversed-transcribed and cDNA was used to determine effects of Pgrmc1 on Ifit3, Gbp4 and Rtp4 mRNA levels. Data were analyzed using Two-Way ANOVA with Pgrmc1 and TNFα as main effects followed by Tukey’s multiple comparison test.

## Results

### Microarray analyses identified several Pgrmc1-regulated functions

**Fig 1:**
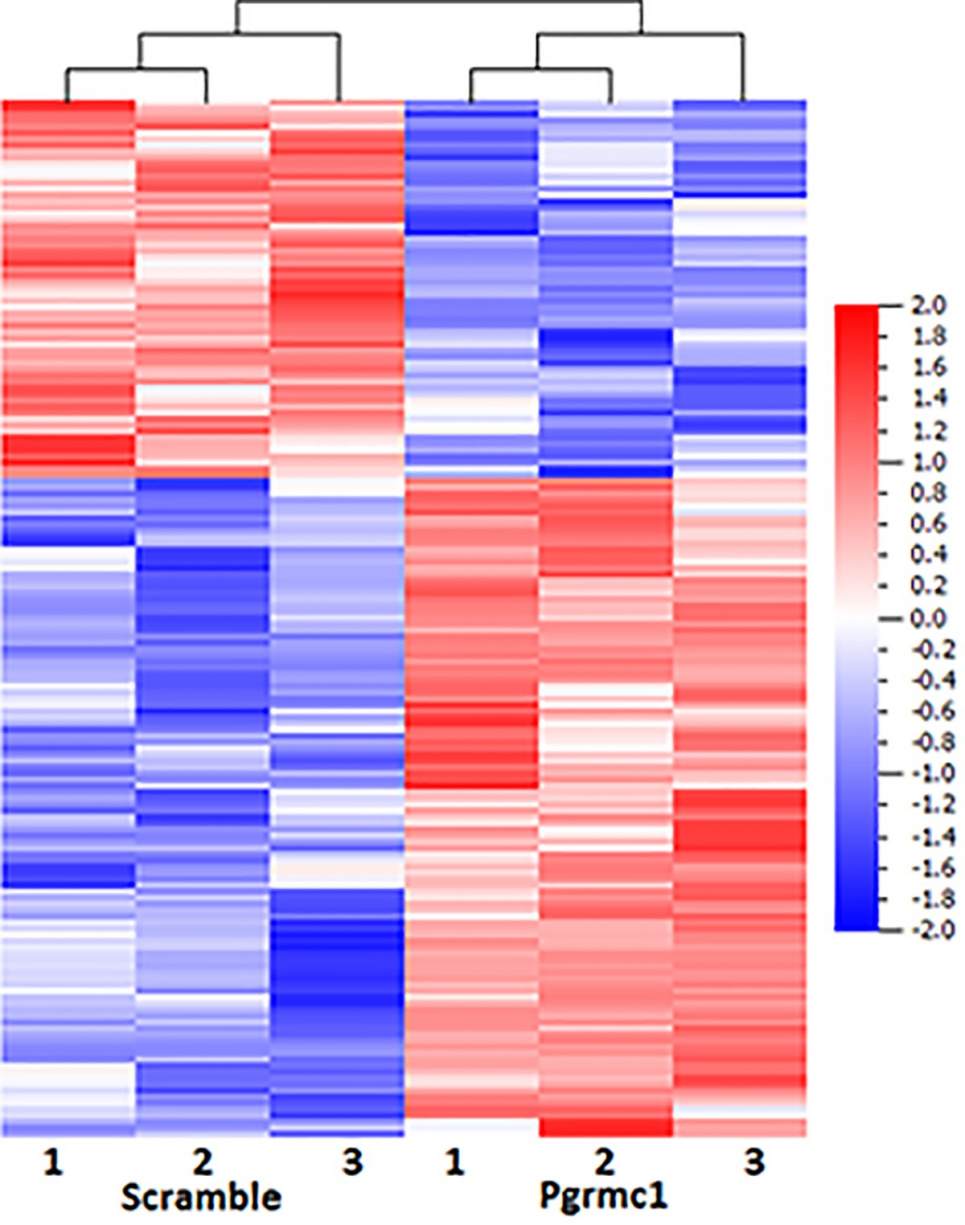
Heat map of microarray data showing relative expression of genes found to be differentially expressed (*p*<0.05) in all three samples for each treatment (scramble siRNA or Pgrmc1 siRNA).

Fig 1 shows the heat map of genes significantly regulated by Pgrmc1 siRNA.

**Fig 2:**
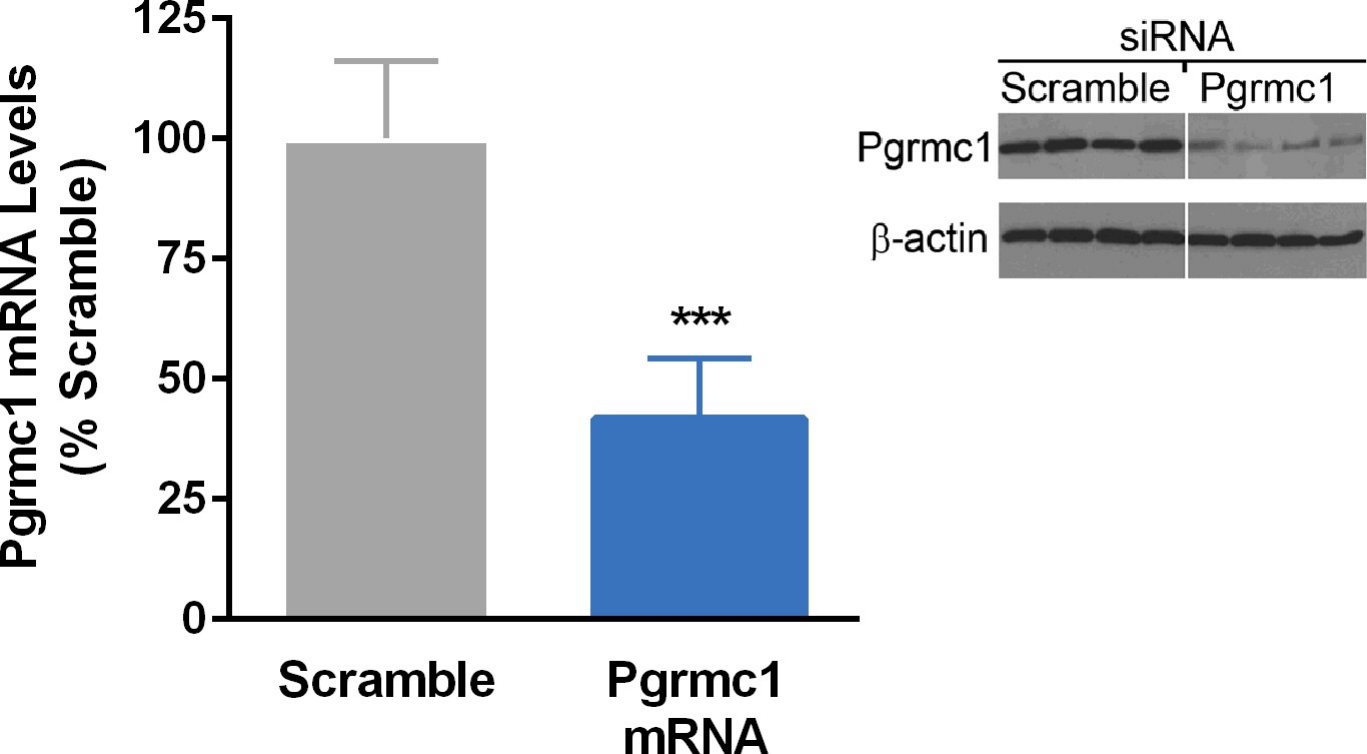
Results of QPCR (left) and western blots (right) verifying knockdown of Pgrmc1 mRNA and protein using siRNA in N42 hypothalamic cells.

Fig 2 shows results of QPCR and western blots showing that Pgrmc1 was knocked down in Pgrmc1 siRNA-treated cells. S1 Dataset provides results of microarray analysis comparing annotated genes in transcriptomes of N42 cells treated with scramble or Pgrmc1 siRNA.

Compared with controls, Pgrmc1 knockdown in the absence of P_4_ changed expression of 1905 annotated genes at the *p*<0.05 level and 590 at *p*<0.01. Expression of eighteen of the identified genes (including *pgrmc1*) changed by at least 1.5-fold with *p*<0.05 (Table 2). Eleven of these genes have been linked to immune/inflammatory response functions, five to Jak/Stat signaling and three to steroid hormone synthesis or signaling (NIH Gene; www.ncbi.nlm.nih.gov/gene and GeneCards; www.genecards.org).

**Table 2:** Genes regulated by at least 1.5-fold and *p*<0.05 in N42 cells transfected with Pgrmc1 siRNA compared with cells transfected with scramble siRNA. Genes in bold have been linked to immune/interferon functions (NIH Gene, www.ncbi.nlm.nih.gov/gene and GeneCards, www.genecards.org).

Gene Name	Gene Symbol	Entrez Gene ID	Fold Change	*P* Value
***Progesterone receptor membrane component 1***	*Pgrmc1*	53328	-2.2	0.00046
***Spectrin, alpha erythrocytic 1***	*Spna1*	20739	-1.5	0.011
***Cell migration inducing protein, hyaluronan binding***	*Cemip*	80982	-1.5	0.040
*Tripartite motif-containing 5*	***Trim5***	**319236**	**1.5**	**0.024**
***Glycoprotein (transmembrane) nmb***	*Gpnmb*	93695	1.5	0.024
*Ubiquitin specific peptidase 18*	***Usp18***	**217109**	**1.5**	**0.002**
***Olfactory receptor, family 51, subfamily B, member 2***	*Or51b2*	18366	1.5	0.044
*Nuclear receptor subfamily 4, group A, member 1*	***Nr4a1***	**15370**	**1.5**	**0.004**
*2'-5' Oligoadenylate synthetase-like 2*	***Oasl2***	**23962**	**1.5**	**0.051**
*Interferon-induced protein 44*	***Ifi44***	**99899**	**1.5**	**0.0001**
*Phospholipid scramblase 2*	***Plscr2***	**18828**	**1.5**	**0.031**
***Ubiquitously-expressed transcript***	*Utx*	22294	1.5	0.001
*Guanylate binding protein 4*	***Gbp4***	**55932**	**1.6**	**0.029**
*Guanylate binding protein 7*	***Gbp7***	**229900**	**1.6**	**0.010**
*Interferon-induced protein with tetratricopeptide repeats 3*	***Ifit3***	**15959**	**1.6**	**0.005**
*T-cell specific GTPase 1*	***Tgtp***	**21822**	**1.7**	**0.007**
***Ets variant 1***	*Etv1*	14009	1.8	0.010
*Receptor transporter protein 4*	***Rtp4***	**67775**	**1.8**	**0.002**

An unbiased GSEA Hallmark study of the 19301 individual genes (S1 Dataset) identified 14 pathways and processes with NOM *p* and FDR q values of *p*<0.05 (Table 3). Nearly half of the pathways and processes were related to Jak/STAT, TNFα, cytokines (interleukins and interferons) and inflammatory regulators (Table 3; shown in bold text). Similarly, results of our Ariadne pathway analysis identified five pathways significantly (*p*<0.05) regulated by Pgrmc1 knockdown and four involved STAT signaling (Table 4).

**Table 3:** Results of GSEA analysis (Hallmark Collection database) of pathways and biological processes that differed significantly between N42 cells transfected with scramble or Pgrmc1 siRNA. Pathways were considered significant if the normalized enrichment score was at least 1.5-fold and nominal P values and false discovery rate Q values were significant (*p*<0.05). Pathways involved in inflammatory processes are shown in bold text.

Pathway or Process Name	Normalized Enrichment Score	Nominal P Value	False Discovery Rate Q Value
Interferon Alpha Response	2.64	0	0
TNFα Signaling via NfkB	2.40	0	0
Interferon Gamma Response	2.31	0	0
E2F Targets	2.15	0	0
Kras Signaling (GTPase)	1.90	0	5.34E-04
Oxidative Phosphorylation	1.89	0	4.45E-04
Myc Targets V1 (transcription/ cell cycle)	1.87	0	3.82E-04
G2M Checkpoint	1.85	0	6.56E-04
DNA Repair	1.84	0	5.83E-04
P53 Pathway	1.75	0	0.0022
Myc Targets V2	1.69	0.004	0.0038
IL6 Jak STAT3 Signaling	1.61	0.010	0.0065
Inflammatory Response	1.57	0	0.010
IL2 STAT5 Signaling	1.51	0	0.017

**Table 4:** Pathways found to contain significantly (*p*<0.05) more Pgrmc1-regulated genes than would be expected by chance. Data were analyzed using Ariadne Pathway Studio Enrichment Analysis.

Rank	Pathway Name	*P-*Value
1	Interleukin 6 Receptor and STAT3 Signaling	0.019
2	Gonadotrope Cell Activation	0.026
3	Urokinase Receptor and STAT Signaling	0.040
4	Interferon α/β Receptor and STAT Signaling	0.041
5	Cilliary Neurotropic Factor and STAT3 Signaling	0.045

Table 5 shows results of our IPA study to identify upstream regulators of the same genes that are altered by Pgrmc1 knockdown. Targets were considered significant if the *p* value of the overlap between dataset genes and genes regulated by a putative upstream regulator was <0.05. A majority of the targets were steroids or molecules related to steroid action or synthesis. In addition, more than half have been linked previously to Pgrmc1 or P_4_, increasing confidence in our data and analyses.

**Table 5:** Top 10 upstream regulators of genes also regulated by Pgrmc1 in N42 hypothalamic cells. Regulators shown in boldface type are steroids, steroid receptors or molecules known to regulate steroid synthesis or activities. Those shown in italic boldface are known to be regulated by Pgrmc1 or P_4_.

Upstream Regulator	Direction of Change	Symbol	Molecule Type	P-Value of Overlap
**β-Estradiol**	**Activated**	**E**_**2**_	**Steroid transcription regulator**	**2.94E-14**
***E2F transcription factor 8***		***E2f8***	***Transcription regulator***	***6*.*94E-10***
**Sterol regulatory element binding factor (Srebf) cleavage-activating protein**	**Activated**	**Scap**	**Chaperone protein**	**8.38E-09**
***Transforming growth factor β1***	***Activated***	***Tgfβ1***	***Cytokine; growth factor***	***1*.*42E-08***
**Huntington**		Htt	Transcription regulator	1.92E-07
**Sterol regulatory element binding factor**	**Activated**	**Srebf2**	**Transcription regulator**	**3.04E-07**
**E2F7 transcription factor 7**		E2f7	Transcription regulator	5.99E-07
***Brain-derived neurotrophic factor***	***Activated***	***Bdnf***	***Nerve growth factor***	***1*.*48E-06***
**Estrogen receptor 1 (encodes estrogen receptor α)**	**Activated**	**Esr1**	**Steroid transcription regulator**	**3.39E-06**
**Harvey rat sarcoma viral oncogene homolog**		Hras	Ras oncogene	3.73E-06

DAVID 6.8 Functional Clustering analysis identified 2112 DAVID IDs and three significantly (*p*<0.05) enriched Annotation Clusters: Guanylate Binding (11.8-fold enrichment), MAPK Activity (1.8-fold enrichment) and SMAD Protein Signal Transduction (1.4-fold enrichment).

### QPCR studies verified microarray findings

**Fig 3:**
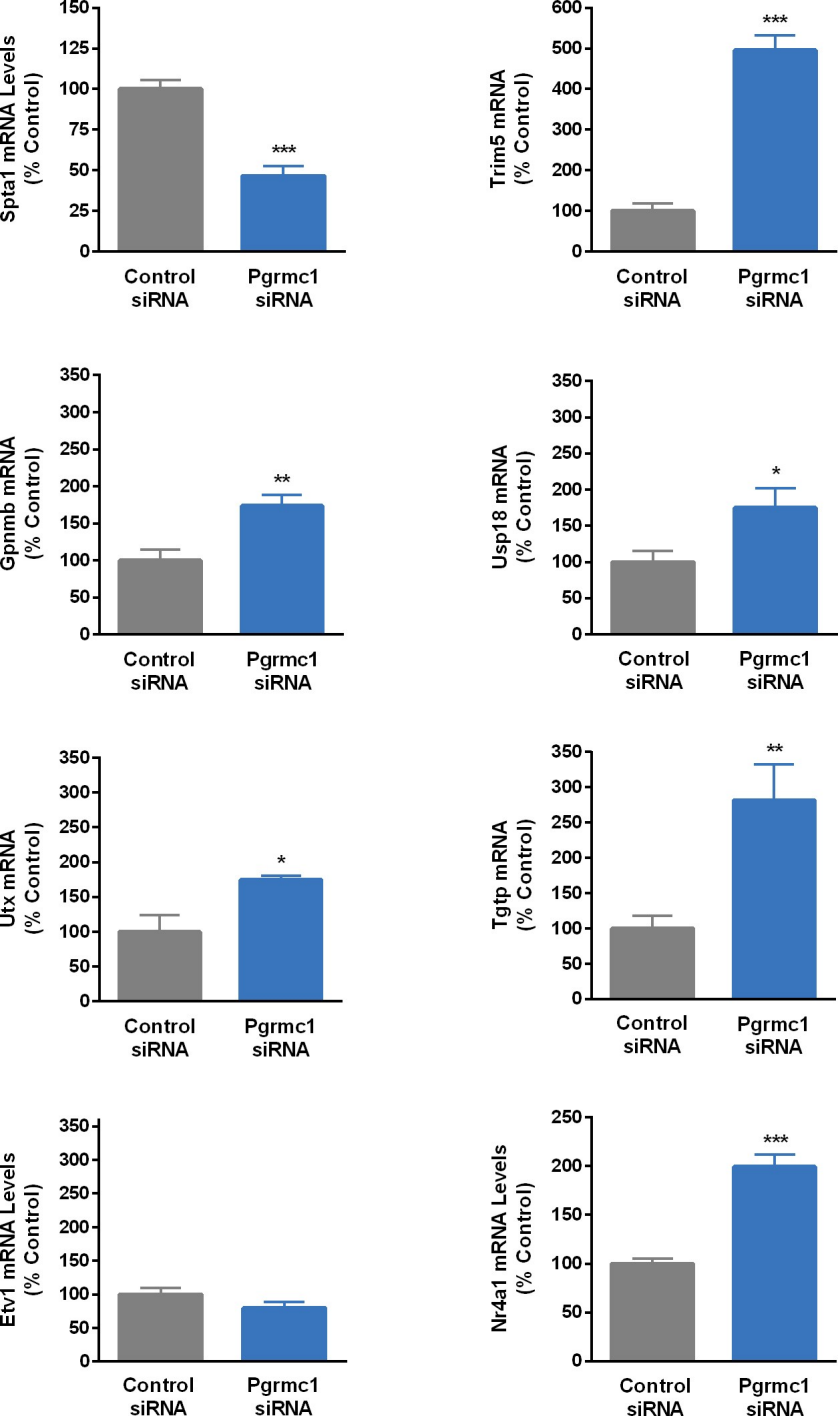
Results of QPCR verification of genes that were found to be regulated by Pgrmc1 knockdown in microarray analysis. N42 cells were transfected with scramble control or Pgrmc1 siRNA. Bars represent mean ± SEM. (*Significantly different from scramble control, *p*<0.05; **p<0.001; ***p<0.0001).

**Fig 4:**
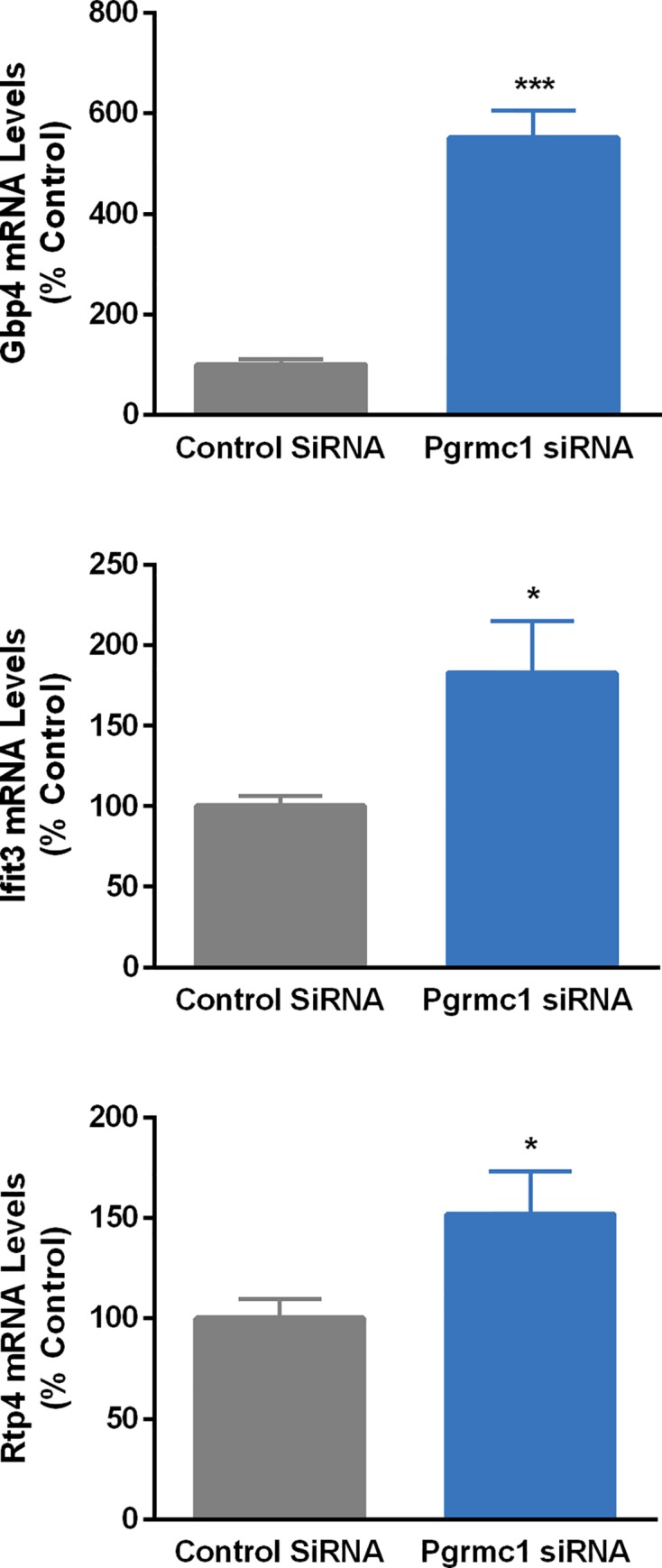
Results of QPCR verification of proinflammatory cytokine gene targets found to be regulated by Pgrmc1 knockdown in microarray analysis. N42 cells were transfected with scramble control or Pgrmc1 siRNA. Bars represent mean ± SEM. (*Significantly different from scramble control, *p*<0.05; ***p<0.0001).

We verified a significant (*p*<0.0001) decrease in Pgrmc1 mRNA levels after treatment of N42 cells with Pgrmc1 siRNA (scramble control = 100.0 ± 4.32; Pgrmc1 siRNA treated = 49.5 ± 4.25). Fig 3 shows results of QPCR studies using RNA from N42 cells treated with Pgrmc1 siRNA. Fig 4 shows similar studies verifying that Pgrmc1 knockdown also upregulates three proinflammatory cytokine gene targets, *gbp4*, *ifit3*, and *rtp4*, that were also among the genes identified as top targets on the microarray. Ten of 11 genes significantly regulated by Pgrmc1 in microarray analysis were verified by independent QPCR validation studies.

### Pgrmc1 receptor antagonist upregulates expression of cytokine target genes

**Fig 5:**
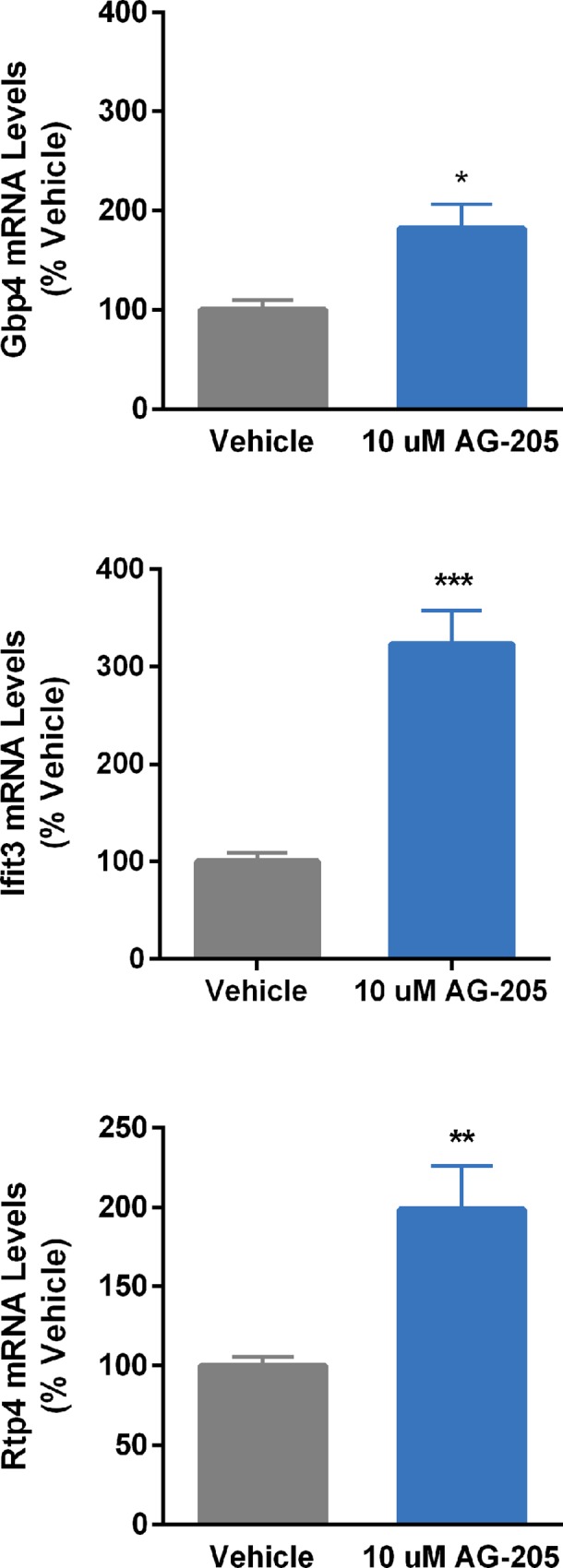
Effects of Pgrmc1 antagonist, AG-205, on genes found to be upregulated by Pgrmc1 knockdown on microarray. N42 cells were treated with 10 μM AG-205 for 24 h before harvesting. Bars represent mean ± SEM. (*Significantly different from vehicle control, *p*<0.05; **p<0.001; ***p<0.0001).

Fig 5 shows that treatment of cells with the Pgrmc1 antagonist, AG-205, upregulates the expression of *gbp4*, *ifit3*, and *rtp4*, verifying that knockdown results were not an artifact of siRNA transfection.

### Pgrmc1 inhibits TNFα-induced upregulation of genes in N42 hypothalamic cells independent of P_4_

**Fig 6:**
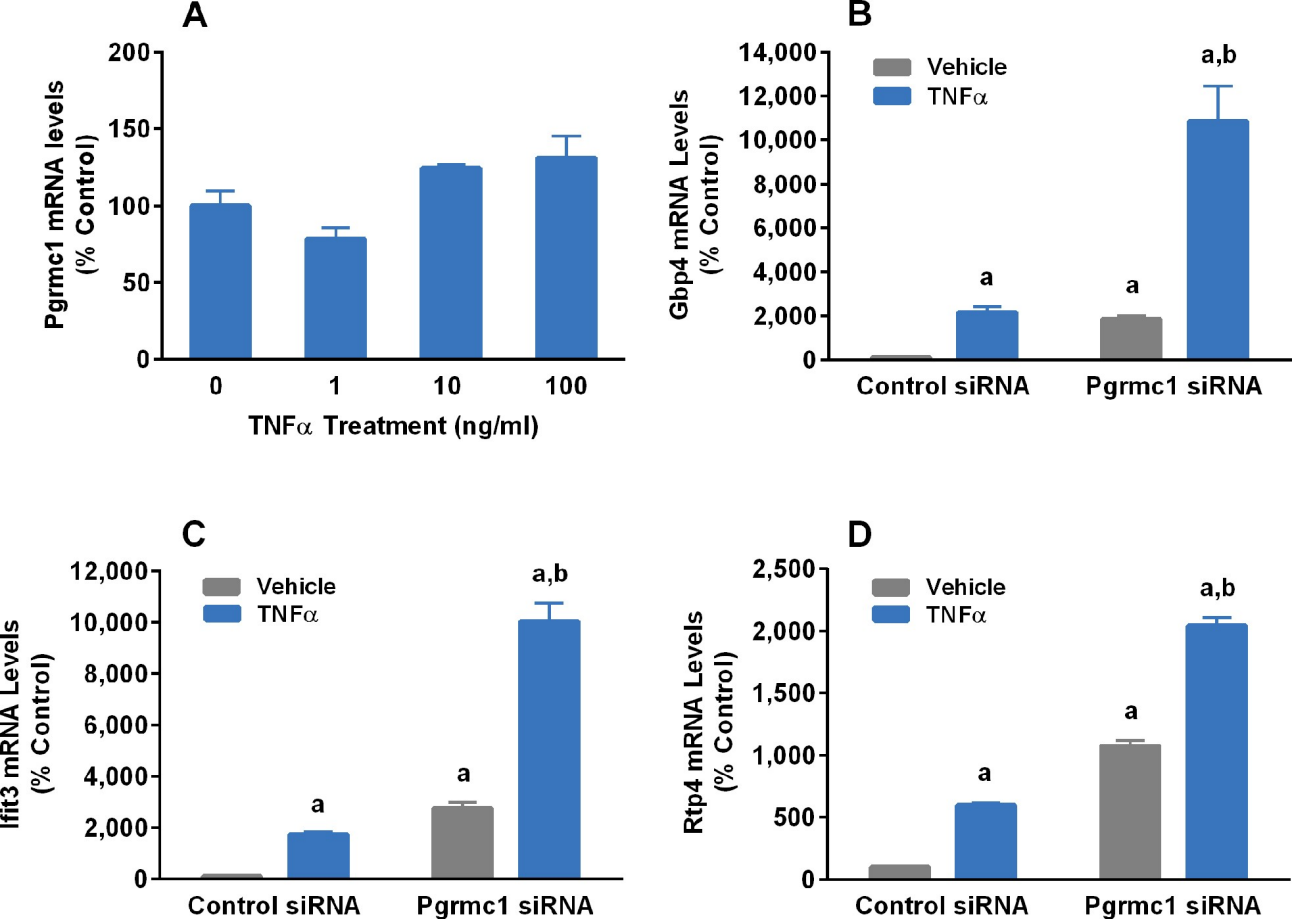
Effects of TNFα on Pgrmc1 mRNA levels (A) and effects of Pgrmc1 knockdown on TNFα (10 ng/ml) regulation of Gbp4 (B), Ifit3 (C) and Rtp4 (D) mRNA levels in N42 cells. Bars represent mean ± SEM. (^a^Significantly different from control cells treated with vehicle, *p*<0.001; ^b^significantly different from cells treated with Pgrmc1 siRNA + vehicle, *p*<0.0001).

**Fig 7:**
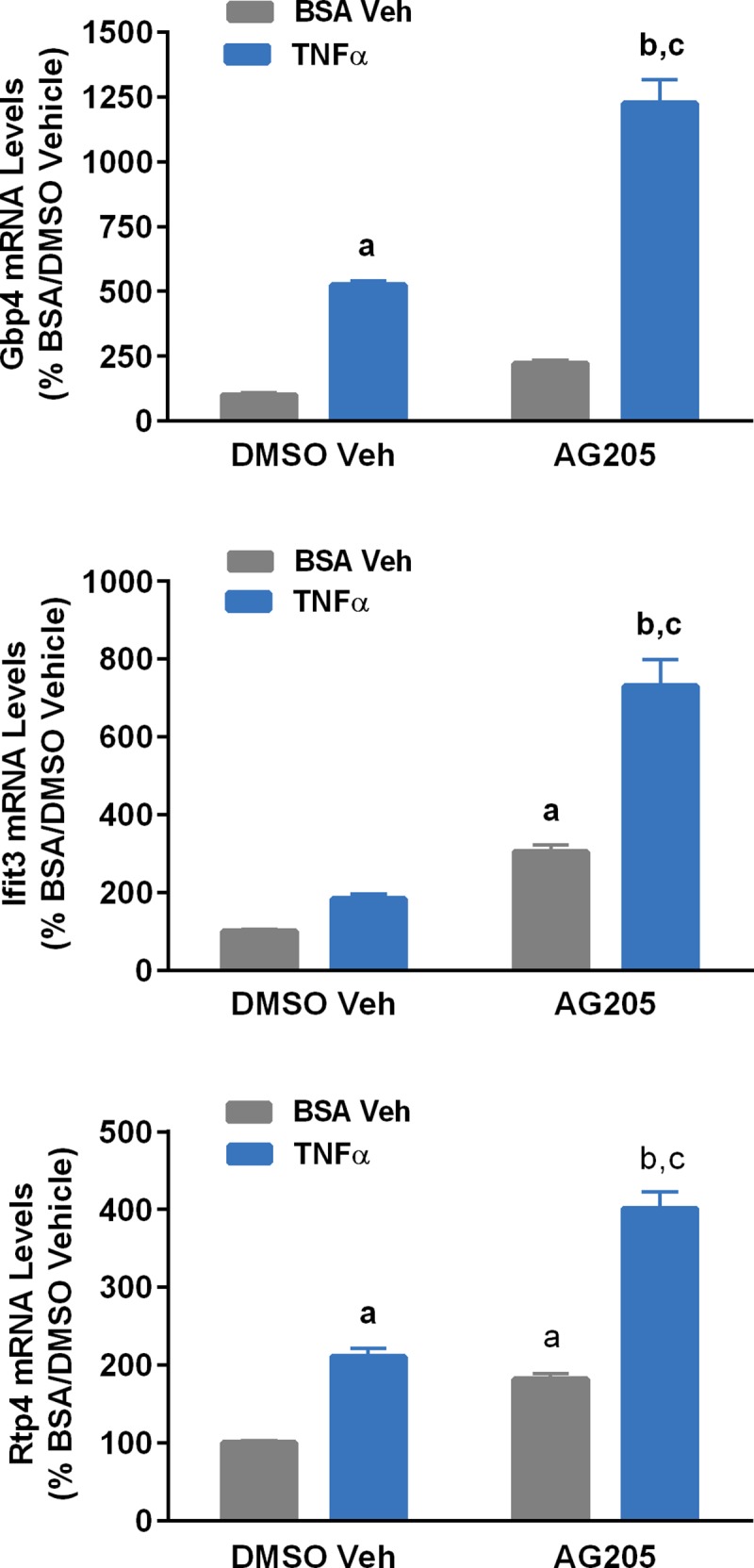
Effects of Pgrmc1 antagonist, AG-205 on TNFα (10 ng/ml) regulation of Gbp4 (top panel), Ifit3 (middle panel) and Rtp4 (bottom panel) mRNA levels in N42 cells. Bars represent mean ± SEM. (^a^Significantly different from control cells treated with DMSO vehicle, *p*<0.0001; ^b^significantly different from cells treated with TNFα + vehicle; ^c^significantly different from cells treated with AG205+vehicle, *p*<0.0001).

Treatment of N42 cells with TNFα did not alter Pgrmc1 mRNA levels; however, Pgrmc1 knockdown significantly increased both basal and TNFα-induced expression of *gbp4*, *ifit3* and *rtp4* (Fig 6). Treatment of cells with the Pgrmc1 antagonist, AG-205, increased basal expression of *ifit3* and *rtp4* and significantly enhanced the ability of TNFα to induce expression of *gbp4*, *ifit3* and *rtp4* (Fig 7).

**Fig 8:**
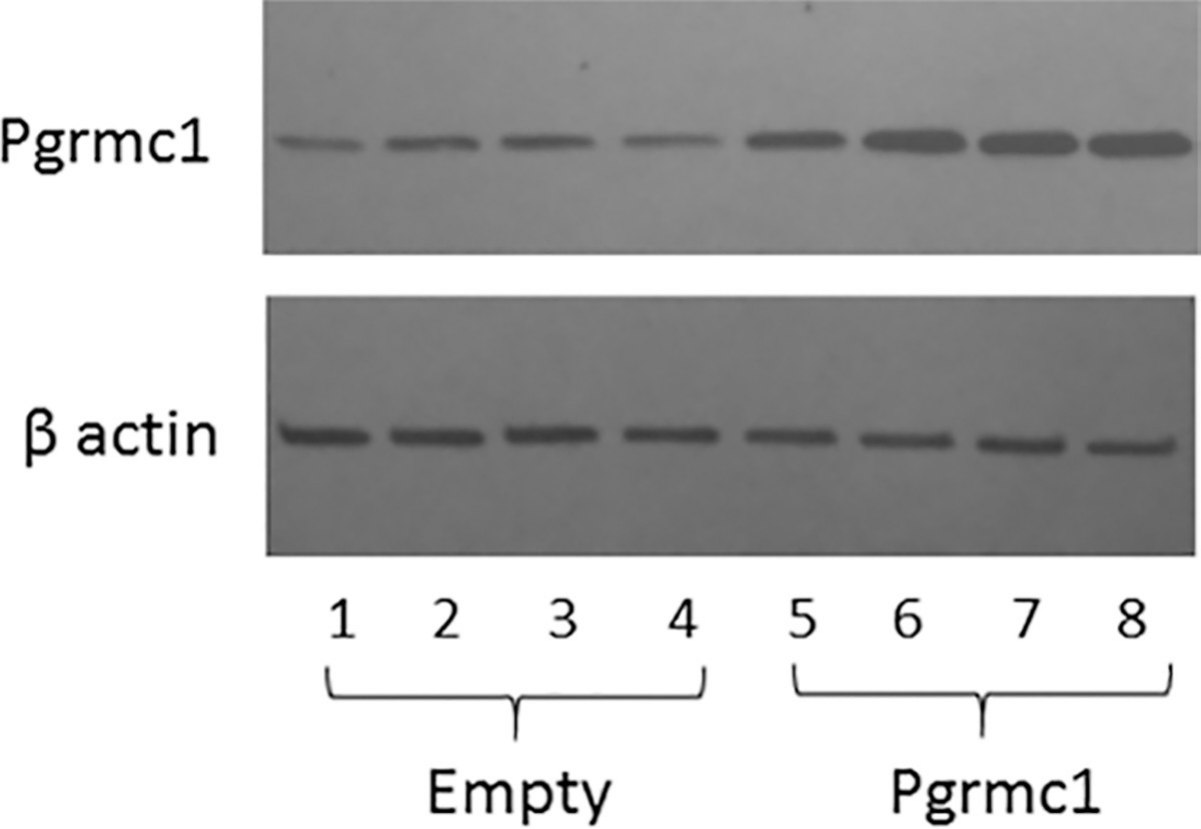
Pgrmc1 protein levels in cells transfected with an empty vector or with a Pgrmc1 expression vector.

**Fig 9:**
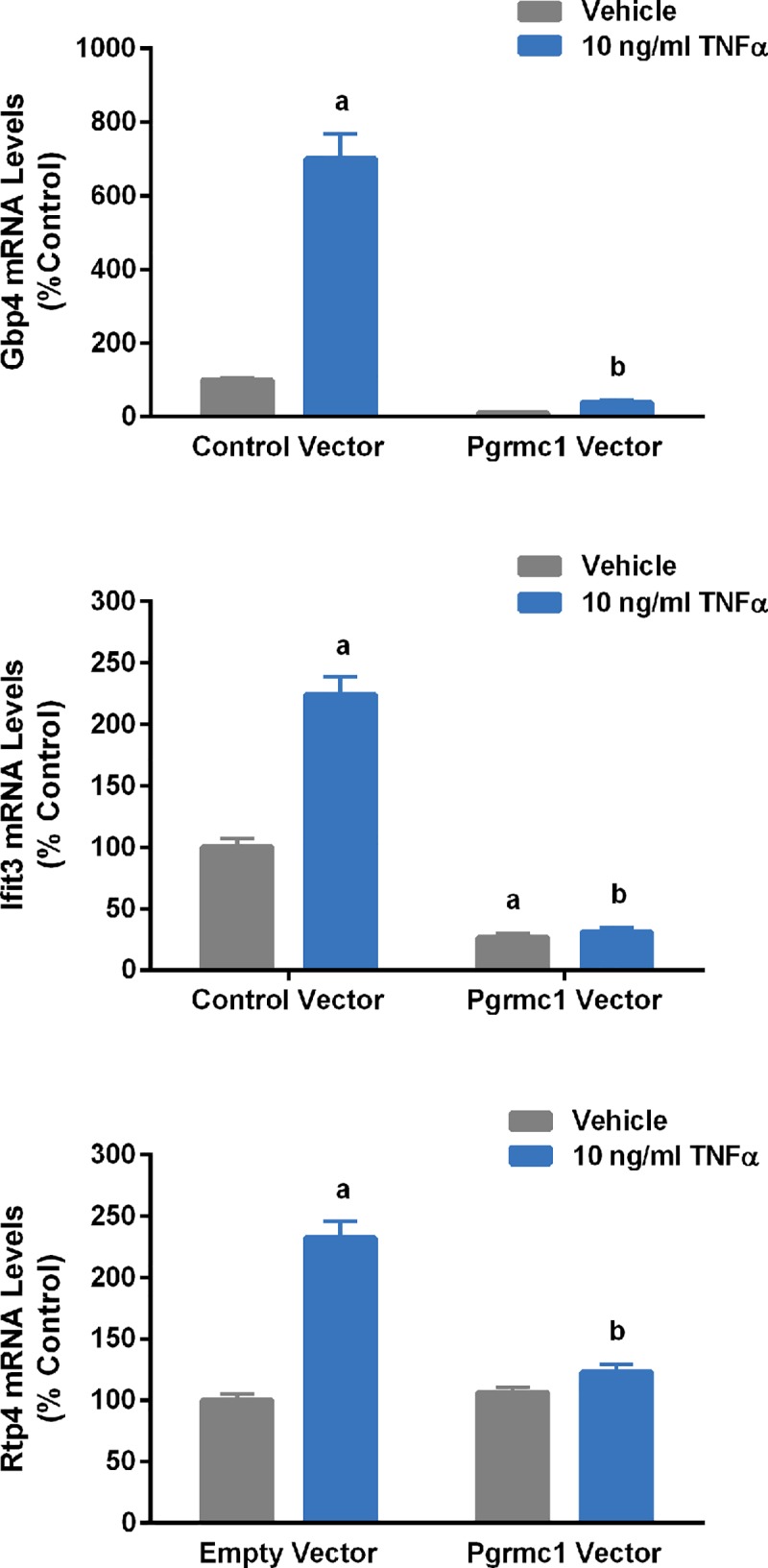
Effects of Pgrmc1 overexpression on TNFα regulation of genes in N42 cells. Bars represent mean ± SEM. (^a^Significantly different from cells with control vector treated with vehicle, *p*<0.0001; ^b^significantly different from cells with control vector treated with TNFα, *p*<0.0001).

**Fig 10:**
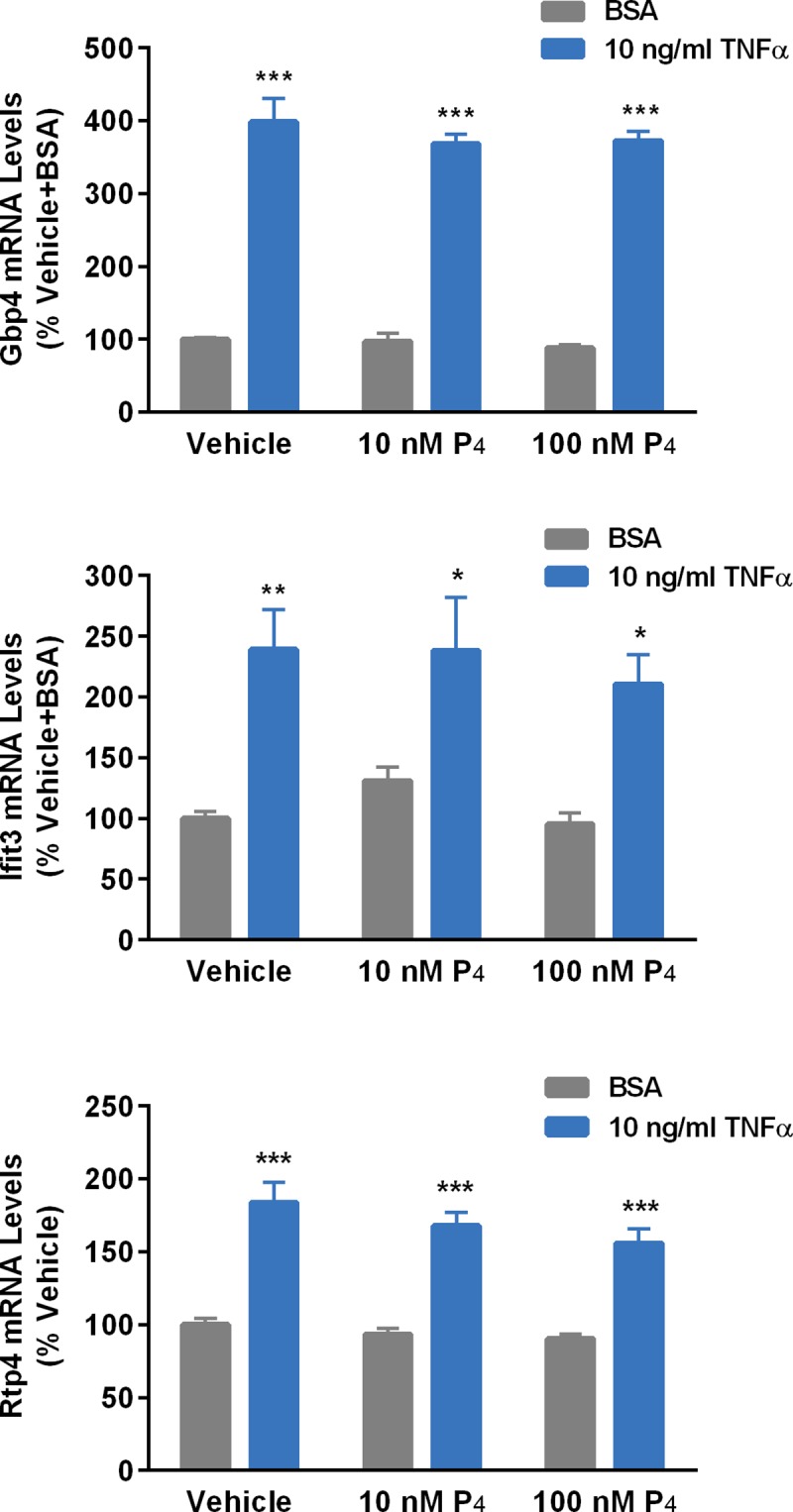
Effects of progesterone (P_4_; 10 or 100 nM) on TNFα (10 ng/ml)-induced upregulation of genes in N42 cells. Bars represent mean ± SEM. (*Significantly different from cells treated with vehicle, *p*<0.05; **significantly different from vehicle-treated, *p*<0.01; ***significantly different from vehicle-treated, *p*<0.0001).

Conversely, Pgrmc1 overexpression in stably transfected N42 cells (Fig 8) completely blocked the ability of TNFα to induce expression of *ifit3*, *gbp4* and *rtp4* genes (Fig 9). P_4_ had no effect on the ability of TNFα to increase the expression of these genes (Fig 10).

### Pgrmc1 inhibits cytokine upregulation of genes in mHippoE-18 hippocampal cells

**Fig 11:**
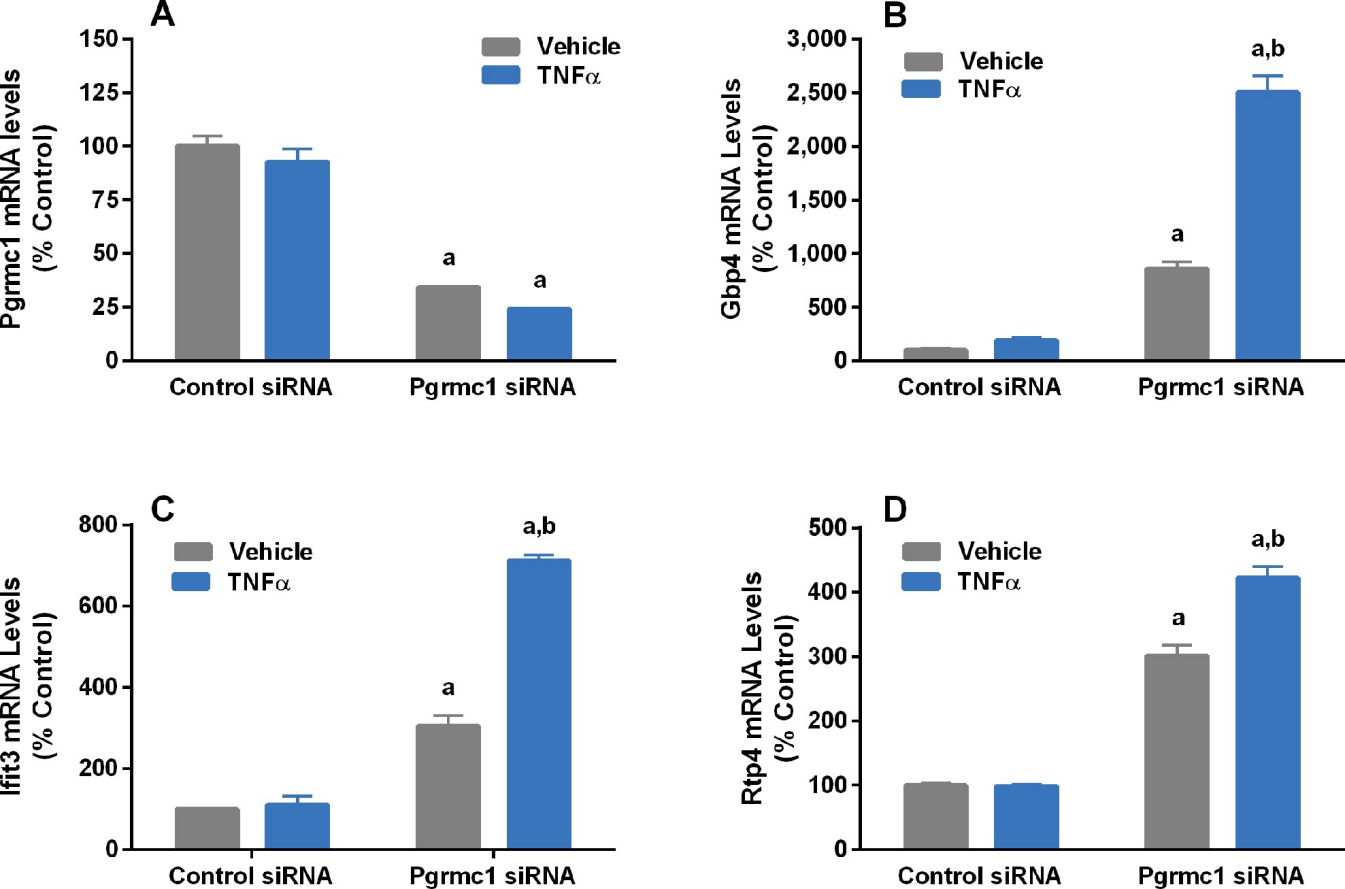
Effects of Pgrmc1 knockdown on TNFα (10 ng/ml) regulation of Pgrmc1 (A), Gbp4 (B), Ifit3 (C) and Rtp4 (D) mRNA levels in mHippoE-18 cells. Bars represent mean ± SEM. (^a^Significantly different from corresponding control cells, p<0.001; ^b^significantly different from cells treated with Pgrmc1 siRNA + vehicle, p<0.001).

As shown in Fig 11, Pgrmc1 knockdown also increased both basal and TNFα-induced expression of *gbp4*, *ifit3* and *rtp4* in hippocampal cells without altering *pgrmc1* expression. However, in contrast to our findings in N42 cells, TNFα did not significantly increase expression of any of these genes in control cells that contained Pgrmc1.

## Discussion

These microarray and bioinformatics findings provide evidence that Pgrmc111 acts independently of P_4_ to regulate signaling important for neuroimmune functions. Among the genes most highly upregulated by Pgrmc1 depletion were *gbp4*, *ifit3*, and *rtp4*, genes induced by proinflammatory cytokines such as interferons, interleukins and Tnfα [30–33]. Our follow-up studies verified that at least one of these cytokines, TNFα, upregulated expression of *gbp4*, *ifit3*, and *rtp4* and expression was inhibited by Pgrmc1 in both hypothalamic and hippocampal cell lines. The effect of Pgrmc1 on the expression of these genes was independent of P_4_, but our analysis of upstream regulators suggests that Pgrmc1 may alter synthesis or signaling of steroids that alter neuroinflammatory signals [34]. Overall, our findings provide new insights into how Pgrmc1 may exert neuroprotective effects.

Pathway analyses of the entire transcriptomes of Pgrmc1 siRNA- and scramble siRNA-treated N42 cells identified pro-inflammatory signaling pathways as regulatory targets of Pgrmc1. In addition, 60% of the most highly regulated genes in our data set were downstream of proinflammatory cytokines. It is unlikely that our findings are due primarily to activation of an innate immune response to siRNA for several reasons. First, *gbp4*, *ifit3*, and *rtp4* were upregulated by a Pgrmc1 antagonist in cells not treated with siRNA. Moreover, basal levels of expression were significantly higher in cells treated with Pgrmc1 siRNA than in those treated with scramble control siRNA. Finally, cells engineered to constitutively overexpress Pgrmc1 had lower levels of Gbp4 and Ifit3 mRNA. Instead, our findings indicate that Pgrmc1 suppresses proinflammatory cytokine signaling in neural cells.

One of the identified cytokines, TNFα, is particularly abundant in hypothalamic and hippocampal regions [35–37] wherein *pgrmc1* expression is also highest [21]. TNFα regulates a wide range of physiological functions controlled by the hypothalamus and hippocampus including sleep [38], food intake [39, 40], learning, memory and anxiety-like behaviors [41]. Moreover, dysregulation of these functions can occur when production of TNFα increases in response to infection or injury [40]. Little is known about Pgrmc1 regulation of these functions under normal conditions, but our findings suggest that Pgrmc1 may mitigate pathological effects of elevated TNFα levels by repressing expression of cytokine effector genes.

A neuropathology that involves TNFα-mediated neuroinflammation and Pgrmc1 signaling is Alzheimer’s disease (AD), a progressive neurological disease that results in cognitive impairment and memory loss. There is some debate about the primary pathophysiology underlying AD. According to the amyloid cascade hypothesis [42], the pathology begins with dysregulation of amyloid beta (Aβ) production and clearance. Aβ deposition, in turn, is thought to disrupt synaptic functions and induce a cascade that eventually produces neuronal loss and deficits in neural transmission underlying impaired memory and cognition. Others argue that, rather than amyloid deposition triggering the disease, it may be a result of neuroinflammation mediated by TNFα [43–45]. Previous work combined with findings reported herein show that Pgrmc1 can impact both Aβ deposition and TNFα signaling. The literature in this area is complicated because Pgrmc1 was thought for several years to be the same molecule as the sigma 2 receptor [46]. It is now clear that they are separate molecules encoded by two different genes [47–49], but both alter Aβ deposition and TNFα signaling. Importantly, Pgrmc1 antibodies or Pgrmc1 siRNA knockdown, as well as antagonists to sigma 2 receptor, prevent Aβ deposition and improve synaptic functioning [50, 51]. Thus, they are logical therapeutic targets for AD treatment. However, such treatments may be complicated because in the present studies we found that Pgrmc1 likely represses TNFα signaling and others showed that sigma 2 agonists inhibit *tnfα* gene expression [52]. Consequently, while Pgrmc1 and sigma 2 antagonists decrease Aβ binding to neurons, they may also block the protective effects of Pgrmc1 and sigma 2 agonists exerted through TNFα inhibition. Further studies will be required to determine how Pgrmc1 and sigma 2 receptor interactions might be manipulated to prevent both Aβ deposition and TNFα-dependent inflammation associated with AD.

The downstream targets of TNFα are not entirely clear in neurons, but our work shows that TNFα upregulates *ifit3*, *gbp4* and *rtp4*, genes among the most highly upregulated when Pgrmc1 was depleted. In addition, both Pgrmc1 depletion and treatment with the Pgrmc1 antagonist, AG-205, enhanced the ability of TNFα to activate expression of *ifit3*, *gbp4* and *rtp4*. Unfortunately, these genes have not been studied extensively in neural cells, so it is difficult to predict how they may affect cellular functions during the inflammatory process. Ifit3 (also known as retinoic acid induced gene G protein) is a Jak-Stat regulated target of interferon and retinoic acid that blocks cell proliferation by upregulating p21 [53]. The other two genes upregulated by TNFα and suppressed by Pgrmc1, *gbp4* and *rtp4* each play a role in G protein signaling. Rtp4 chaperones G-protein coupled receptors to the cell surface [54, 55] and Gbp4 hydrolyzes GTP to both GDP and GMP [56]. Rtp4 also increases trafficking of mu-delta heterodimeric opioid receptors to the cell surface, thereby changing the cellular responses to endogenous ligands [55]. Both mu and delta opioid agonists repress TNFα production [57, 58], suggesting a possible feedback mechanism in the system. Further studies are necessary to determine what role these genes might play in neuroinflammation.

Although P_4_ did not affect basal or TNFα induction of Pgrmc1 target genes, one interpretation of our bioinformatics data is that Pgrmc1 indirectly exerts neuroprotective effects by modulating neurosteroid synthesis or signaling. Interrogation of the entire Pgrmc1-dependent transcriptome without considering fold-change determined that over half of the top 20 upstream regulatory pathways identified involved steroid signaling or synthesis. It was beyond the scope of this paper to examine whether steroidogenesis occurs in N42 cells or if Pgrmc1 might alter the process, but previous work shows that Pgrmc1 directly binds to and activates cytochrome P450 enzymes critical for steroid synthesis [1, 2, 4, 59]. In addition, we found that Pgrmc1 regulates expression of *nr4a1* (also known as *nur77*, *tr3*, *nak-1 and ngf1b*), an immediate early gene required for the induction of a number genes encoding steroidogenic enzymes [60–63]. Thus, an important question to explore in future studies is whether Pgrmc1 alters synthesis of neurosteroids including progesterone, estrogen or steroid metabolites that are neuroprotective [63].

In summary, our findings suggest that Pgrmc1 may exert neuroprotective effects by blocking TNFα induction of downstream gene targets including *gbp4*, *ifit3*, and *rtp4*. In addition, Pgrmc1 may alter neuroinflammation by regulating neurosteroid synthesis, both directly through steroidogenic enzyme activation and indirectly through *nr4a1* regulation of steroidogenic enzyme expression. Our findings provide important new information about actions of Pgrmc1 in neural cells, and suggest new areas for exploration in the search for therapeutic targets to better treat neurodegenerative diseases.

## Supporting information

S1 Dataset Full set of annotated genes derived from microarray analysis comparing transcriptomes of N42 cells treated with scramble or Pgrmc1 siRNA. This dataset was used for GSEA analysis.
(XLSX)

S2 Dataset Genes found to change by 1.2-fold with p<0.05 in microarray analysis comparing transcriptomes of N42 cells treated with scramble siRNA or Pgrmc1 siRNA. This dataset was used for Ingenuity Pathway analysis of upstream regulators and for DAVID analysis.
(XLSX)

S1 Fig Photomicrograph of PCR analysis using primers to Pgr (Forward, CTCCGGGACCGAACAGAGT; Reverse, ACAACAACCCTTTGGTAGCAG) and cDNA reverse transcribed from mRNA obtained from anteroventral periventricular nucleus of female mice (lanes 1–3), lysates of N42 cells (lanes 4–6) or run with no template (Lane 7). Both animals and cells were treated with estradiol to maximize Pgr expression.
(TIF)
