# Supplementary figures and images for: Progesterone receptor membrane component 1 inhibits tumor necrosis factor alpha induction of gene expression in neural cells
Source: PLoS One. 2019 Apr 26;14(4):e0215389. doi: 10.1371/journal.pone.0215389 (PMC6485904; doi:10.1371/journal.pone.0215389)

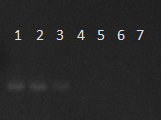

Supplement: S1 Fig — Both animals and cells were treated with estradiol to maximize Pgr expression. (TIF) [file pone.0215389.s003.tif]
